# An Ambiguous Role for Fever in Worsening Outcome After Intracerebral Hemorrhage

**DOI:** 10.1007/s12975-022-01010-x

**Published:** 2022-04-02

**Authors:** Lane J. Liddle, Christine A. Dirks, Mohammed Almekhlafi, Frederick Colbourne

**Affiliations:** 1 Department of Psychology, University of Alberta, Edmonton, AB Canada; 2 Calgary Stroke Program, Calgary, AB Canada; 3 Neuroscience and Mental Health Institute, University of Alberta, P217 Biological Sciences Building, Edmonton, AB T6G 2E9 Canada

**Keywords:** Intracerebral hemorrhage, Meta-analysis, Fever, Hyperthermia, Translational research

## Abstract

Intracerebral hemorrhage (ICH) accounts for 10–15% of all strokes and leaves most survivors with impairments. Fever, a rise in the thermoregulatory set point, complicates ICH. This review summarizes ICH fever studies and employs meta-analytic techniques to explore the relationship between fever and ICH. We discuss methodological considerations for future studies and provide an overview of mechanisms by which fever, and its treatment, may impact ICH. We searched the PubMed database using the following terms: ((fever OR hyperthermia) AND (intracerebral hemorrhage OR intraparenchymal hemorrhage OR intracerebral haemorrhage OR intraparenchymal haemorrhage)). Our search returned 727 studies, of which 21 were included in our final analysis, consisting of 19 clinical, and two preclinical, studies. We conducted a meta-analysis on the clinical data to quantify how fever is related to mortality, functional outcomes, and intraventricular hemorrhage. Analysis of clinical studies suggested that fever causes an increased risk of mortality but does not appear to be associated with poor outcomes among survivors, making it difficult to ascertain the extent of harm caused by post-ICH fever or the benefits of its treatment. Perhaps these inconsistencies stem from variable fever definitions, and temperature measurement and fever treatment protocols. Additionally, the lack of mechanistic data in clinical studies coupled with preclinical studies showing no harmful effects of moderate bouts of hyperthermia raise concerns about the direct contribution of hyperthermia and fever in post ICH outcome. Overall, the significance of temperature increases after ICH is unclear, making this an important area for future research.

## Introduction

Intracerebral hemorrhage (ICH), characterized by cerebral vascular rupture and parenchymal bleeding, is a medical emergency with high death and disability rates [1]. There are currently no clinically approved neuroprotective therapies to improve outcome despite the fact that ICH has many deleterious processes such as mechanical injury, altered cerebral blood flow and metabolism (e.g., potentially ischemia), excitotoxicity, neurotoxicity, and excess inflammation, among many other contributors [2–4]. Despite considerable advances in understanding post-ICH injury pathways, some areas remain poorly understood or controversial. Notably, our understanding of the pathophysiology of ICH is weaker than that of cerebral ischemia, where the relationship between injury and subsequent changes in brain temperature is better understood [3, 5, 6]. In preclinical studies, body temperature measurements are often used as a proxy measure for brain temperature because brain temperature measurements are invasive, making brain temperature measurements relatively uncommon [7]. In clinical studies, many different temperature measurement sites are used (e.g., axillary, rectal, tympanic). Body temperature correlates reasonably with brain temperature measurements in healthy individuals, but brain temperature is known to decouple from body temperature following brain injury [8, 9]. Unfortunately, brain and body temperature increases after ICH (e.g., as a consequence of fever) have received modest attention, and it is unknown if such temperature increases worsen injury following ICH. Research into understudied disease processes such as fever may yield therapeutic targets and improve outcomes.

Human body temperature normally fluctuates around 37 °C, but can vary in healthy individuals by ~ 1 °C, depending on a variety of factors, such as time of day, age, and sex [10–12]. Fever is often arbitrarily defined using temperature threshold cut-offs (e.g., body temperature ≥ 38.3 °C), but physiologically, fever is defined as an increase in body temperature due to an increase in the thermoregulatory set-point of the body [13]. This thermoregulatory set point is homeostatic (i.e., the body is at a physiologically optimal temperature, and the organism actively maintains that temperature; [14]). In contrast, hyperthermia is defined as an elevated body temperature without a set point change, and thus may arise from external temperature changes such as increases in energy production (e.g., physical activity) or imbalances in temperature regulation processes. Simply put, a set-point change means that organism will maintain a temperature that is different than a previous set-point (e.g., the body will engage heat production processes in order to transition to its new set point during fever), whereas a body temperature change without a set-point change involves the organism actively maintaining the initial set-point (i.e., during hyperthermia, sweating and heat loss mechanisms will be engaged). In the context of brain injury, differentiating hyperthermia and fever is critical as mechanisms of injury and therapeutic targets may differ. Fever develops as a consequence of damage to thermoregulatory nuclei, or in response to medication, such as phenytoin; [15]), or pyrogens — either exogenous (e.g., bacterial, fungal, or viral) or endogenous (i.e., produced by the body) [16]. Fever after brain injury can be of neurogenic and/or infectious origin. Accurately determining the etiology of fever is essential, as misdiagnoses may lead to ineffective treatments and sub-optimal patient outcomes.

Neurogenic fever is observed in up to 37% of brain injured patients and is predicted by frontal lobe injury, subarachnoid or ventricular presence of blood, large aneurysms, and decreased level of consciousness [17]. Ventricular extension of the hematoma is relatively common in ICH, affecting ~ 40% of patients [18–20], and given the anatomical proximity of hypothalamic thermoregulatory nuclei to the ventricular system, intraventricular hemorrhage is a key risk factor for neurogenic fever in ICH [21]. Infectious fever is also important to consider, as bacterial infections are relatively common in ICH patients and can lead to complications such as pneumonia and sepsis [22]. The commonality of bacterial infections arises possibly, in part, because of stroke-induced immunosuppression [23]. Post-ICH hyperthermia is rarely described in the literature, but it may occur in some circumstances (e.g., cerebral hypermetabolism as a result of abnormal post-ICH brain activity) [24]. Unfortunately, hyperthermia and fever are often used interchangeably, making estimates of hyperthermia difficult to ascertain and thus further study on the incidence and risk factors for post-ICH hyperthermia is required. Overall, fever (or hyperthermia) can impact between 25 and 91% of ICH patients [25, 26].

In this article, we systematically review preclinical and clinical literature surrounding the role of fever in ICH, describe mechanisms through which post-ICH injury may be worsened by fever and risks associated with treating post-ICH temperature increases, and conclude with directions for future research in this domain. Referring to the most recent guidelines for management of spontaneous ICH, it is clear that the role of fever in ICH is uncertain and that further research is required [6, 27].

## Methods

### Literature Search

We searched the PubMed database on January 21, 2021, for English-language studies relating to fever and ICH using the following terms: ((fever OR hyperthermia) AND (intracerebral hemorrhage OR intraparenchymal hemorrhage OR intracerebral haemorrhage OR intraparenchymal haemorrhage)). We screened all returned abstracts and constructed our own literature database of studies relating to fever and ICH. Only primary literature relating to fever and ICH were included in full-text analysis and data extraction. All study types were considered. We did not include studies assessing the effectiveness of antipyretic treatments as a primary endpoint. We re-ran the initial search on November 5, 2021, using the same terms and combined the results of the searches.

We also conducted an exploratory analysis on literature related to the use of antipyretics on fever after ICH. This additional search was conducted on November 22, 2021, for English-language studies only using the following terms: ((fever OR hyperthermia) AND (intracerebral hemorrhage OR intraparenchymal hemorrhage OR intracerebral haemorrhage OR intraparenchymal haemorrhage) AND (antipyretic OR fever management OR fever prevention OR normothermia)). Of the 211 studies returned, 73 appeared relevant and underwent abstract screening, of which 11 remained for full-text analysis and data extraction. Only clinical studies were included. Literature reviews and abstract submissions were excluded.

### Study Quality Assessment

We gathered data that may illustrate the quality of current literature, address current limitations, and suggest future directions for studies exploring fever and ICH. Factors considered for quality assessment were as follows: study type, stroke type (e.g., strictly ICH), temperature monitoring, definition of fever, and incidence of fever. We also tabulated all studies regarding the use of antipyretic agents in ICH.

### Meta-analysis of Endpoints Related to Post-ICH Fever

Meta-analysis was conducted to obtain a quantitative understanding of the relationship between fever and patient outcomes. In particular, we investigated whether intraventricular extension of the hematoma (identified by magnetic resonance or computed tomographic imaging) increased the likelihood of fever, and investigated whether fever increased the likelihood of poor functional outcomes (measured using the modified Rankin scale) or mortality. Odds ratios were gathered across studies and combined using the restricted maximum-likelihood estimator method; this estimator is advantageous when there are few studies returned during a meta-analysis [28]. Adjusted odds ratios were sought and combined wherever possible; however, unadjusted odds ratios were more common. Adjusted and unadjusted odds ratios were not combined. We also constructed funnel plots and performed Egger regression to visually and formally analyze publication bias. Given the small number of studies and heterogeneity in the clinical ICH literature, we opted to perform random effects analysis over fixed effects analysis, because fixed effects analyses can result in narrower confidence intervals and may produce greater Type I error rates than random effects analyses [29]. Given the small number of studies, meta-regression could not be used to explore the relationship between study parameters (e.g., fever definition or duration) and outcomes of interest. All data were analyzed using R statistical software (version 4.1.2, Vienna, Austria).

## Results

### Literature Search

**Fig. 1:**
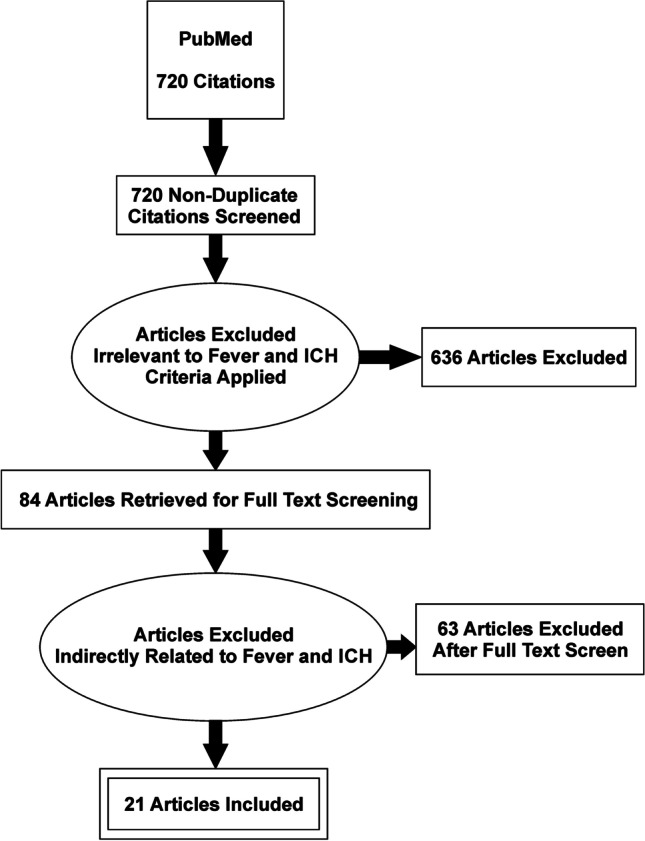
Preferred Reporting Items for Systematic Reviews and Meta-Analyses (PRISMA) diagram of systematic search of English-language literature with the following search criteria: ((fever OR hyperthermia) AND (intracerebral hemorrhage OR intraparenchymal hemorrhage OR intracerebral haemorrhage OR intraparenchymal haemorrhage))

Our search criteria returned 694 articles, and after title and abstract screening, 79 studies appeared to be relevant and underwent full text screening (Fig. 1). Upon screening, only 20 studies were included for data extraction. Studies were then categorized as clinical (18) or preclinical (2). Data from both searches were combined (Fig. 1). The November 5 search yielded 33 more papers, leaving one additional clinical paper to be included for data extraction.

Our additional search on the use of antipyretic agents after fever in ICH returned 211 articles, and after title and abstract screening, 11 studies remained for full text analysis.

### Preclinical Studies

Currently, only two preclinical studies have examined the effects of hyperthermia after ICH. Both studies were conducted in the same laboratory, used young-adult male rats and assessed some mechanism of action regarding the role of hyperthermia post-ICH (e.g., edema, inflammatory markers). Ultimately, both preclinical ICH studies suggest that hyperthermia does not, or only transiently affects, post-ICH outcomes.

The first study employed the collagenase model of striatal ICH that produced a 60-mm^3^ lesion on day 7 post-ICH (a large injury in rat), and induced hyperthermia with an infrared lamp to either 38.5 °C from 1 to 24 h or 24 to 48 h post-ICH, or to 40 °C for 3 h beginning 12 h post-ICH [30]. Brain and body temperatures were monitored via implantable telemetry probes. Here, the authors found that hyperthermia does not aggravate functional outcomes on post-ICH day 2, 7, or 30 (measured using a neurological deficit scale, the Montoya staircase test, ladder walking test, or the cylinder test) or lesion volume on post-ICH day 7, or 30. Bleeding did not appear to be influenced by hyperthermia, as assessed by a spectrophotometric hematoma volume assay, nor did hyperthermia affect the number of activated macrophages (assessed using Perls’ staining).

The second study employed the autologous whole-blood model of ICH (100 μL into striatum, which is a commonly used hematoma volume) and used a heating pad and infrared lamp to raise temperature to 39 °C for 3 h immediately post-ICH [31]. Brain and body temperatures were monitored with thermocouple and rectal probes, respectively. Here, the authors unexpectedly found that hyperthermic animals had better functional outcomes at 24, 48, and 72 h post-ICH (assessed using a neurological deficit scale), and better performance on the horizontal ladder test 72 h post-ICH. These effects were not maintained at 32 days. The authors noted these temporary improvements may be, in part, due to concurrent reductions in edema at 72 h post-ICH. Given the findings of this study, the authors concluded that brief hyperthermia following whole-blood ICH provides transient, modest benefit early after ICH with respect to edema and behavioral outcomes that did not influence later histological or behavioral outlook.

It is important to note that these two studies did not observe spontaneous fever in these ICH models, which is in line with many other ICH studies using models of striatal ICH [32, 33], nor did they induce fever or apply antipyretics. Accordingly, animal models are limited to producing hyperthermia in ICH with physical heating. Notably, physical heating paradigms (i.e., hyperthermia) differ from the development and progression of fever in patients, which would influence subsequent therapeutic approaches. Nonetheless, they did induce hyperthermia to a level previously shown to markedly worsen ischemic damage in rats [34]. Despite that, no harmful effects were observed in ICH. Further, little attention has been paid to age and comorbidity effects in the animal literature, despite their significant impacts on outcome [35, 36]. Future research is needed to investigate this point further.

### Clinical Studies

Of the 19 clinical articles returned for full-text review, 68% only included patients with ICH, 84% monitored temperature(s) (e.g., brain and/or body), 79% reported where temperature measurements were taken, 95% stated their definition of fever, 74% reported the incidence of fever, and 58% employed antipyretic treatment(s) and reported antipyretic treatment protocols (Table 1). Study types were retrospective (9), prospective (9), or case-controlled (1). Conclusions on common endpoints (e.g., ICH size, location) were relatively similar across all studies; that is, authors concluded that fever worsened outcomes. The average (± SD) hematoma volume across all studies was 37.30 mL + / − 44.93 mL. Febrile patients had an average hematoma volume of 61.37 mL + / − 60.31 mL compared to 33.95 mL + / − 33.47 mL for patients without fever. Studies that separated hematoma volume by good and poor outcomes on the Modified Rankin Scale (mRS) yield a combined weighted average of 19.06 mL + / − 19.20 mL for good outcome and 52.1 mL + / − 39.45 mL for poor outcome. It seems that growth may occur in tandem with intraventricular extension, possibly more so for those with fever. Clinical studies employed various definitions of fever across studies (e.g., > 37.5 °C up to ≥ 38.8 °C). Of the studies that reported the incidence of fever, estimates ranged from 1.4 to 69%, with a mean incidence of ~ 35%. While it seems that most studies reported fever incidence upon admission, it is difficult to discern the time course of fever due to broad or unclear definitions (e.g., how many temperature measurements exceeding a threshold are required to be considered febrile). Relationships between ICH location and fever incidence are also difficult to interpret as locations were often pooled and analyzed together. We found no correlation between the definition of fever used in a study and fever incidence estimates (r =  − 0.07; *p* = 0.8). The most common site that authors used to measure body temperature was the axilla (6 studies; 33%). Rectal, oral, and tympanic measurements were less common (22% for each). Seven studies did not clearly state the measurement site. Eleven studies described their protocol to treat fever, with eight studies employing acetaminophen (often 500–1000 mg oral; mostly in line with established guidelines) as a first-line of defense against fever [37, 38]. Five studies also described additional surface cooling measures to combat high or persistent fever, one study used antibiotic treatment for fever, and two studies mentioned pharmacological control of fever with unspecified antipyretics. None of these studies tested whether antipyretics or other temperature control methods affected outcomes (e.g., temperature responses, modified Rankin scores, mortality, etc.).

**Table 1:** Summary of basic study parameters reported in clinical studies (✓ = yes, ✕ = no)

Reference	Strictly ICH	Continuous temperature monitoring	Temperature measurement site	Definition of fever	Incidence of fever (%)	Fever treatment protocol	Employed antipyretic agents
Blanco et al., 2012	✕	✓	✓	✓	✕	✓	✓
Boysen et al., 2001	✕	✓	✓	✓	✕	✓	✓
Bush et al., 2018	✓	✓	✓	✓	✓	✓	✓
Campos et al., 2013	✕	✓	✓	✓	✓	✓	✓
Deogaonkar et al., 2005	✓	✓	✓	✓	✓	✕	✕
Gillow et al., 2017	✓	✓	✓	✓	✓	✕	✕
Halthore et al., 2010	✕	✓	✓	✓	✓	✓	✓
Hervella et al., 2020	✓	✓	✓	✓	✓	✓	✓
Honig et al., 2015	✓	✓	✓	✓	✓	✓	✓
Iglesias-Rey et al., 2018	✓	✓	✓	✓	✓	✕	✕
Leira et al., 2004	✓	✓	✕	✓	✕	✓	✓
Malavera et al., 2021	✓	✕	✕	✓	✓	✕	✕
Naidech et al., 2009	✕	✓	✕	✓	✕	✓	✓
Niphon Poungvarin et al., 2006	✓	✕	✓	✓	✓	✕	✕
Rincon et al., 2013	✓	✓	✓	✓	✓	✕	✕
Roy et al., 2004	✕	✓	✓	✓	✓	✕	✕
Schwarz et al., 2000	✓	✓	✓	✓	✓	✓	✓
Suzuki et al., 1995	✓	✕	✕	✕	✕	✕	✕
Szczudlik et al., 2002	✓	✓	✓	✓	✓	✓	✓
Total (%)	68	84	79	95	74	58	58

### Analyses of the Relationship Between Fever and Post-ICH Outcomes in clinical studies 

**Fig. 2:**
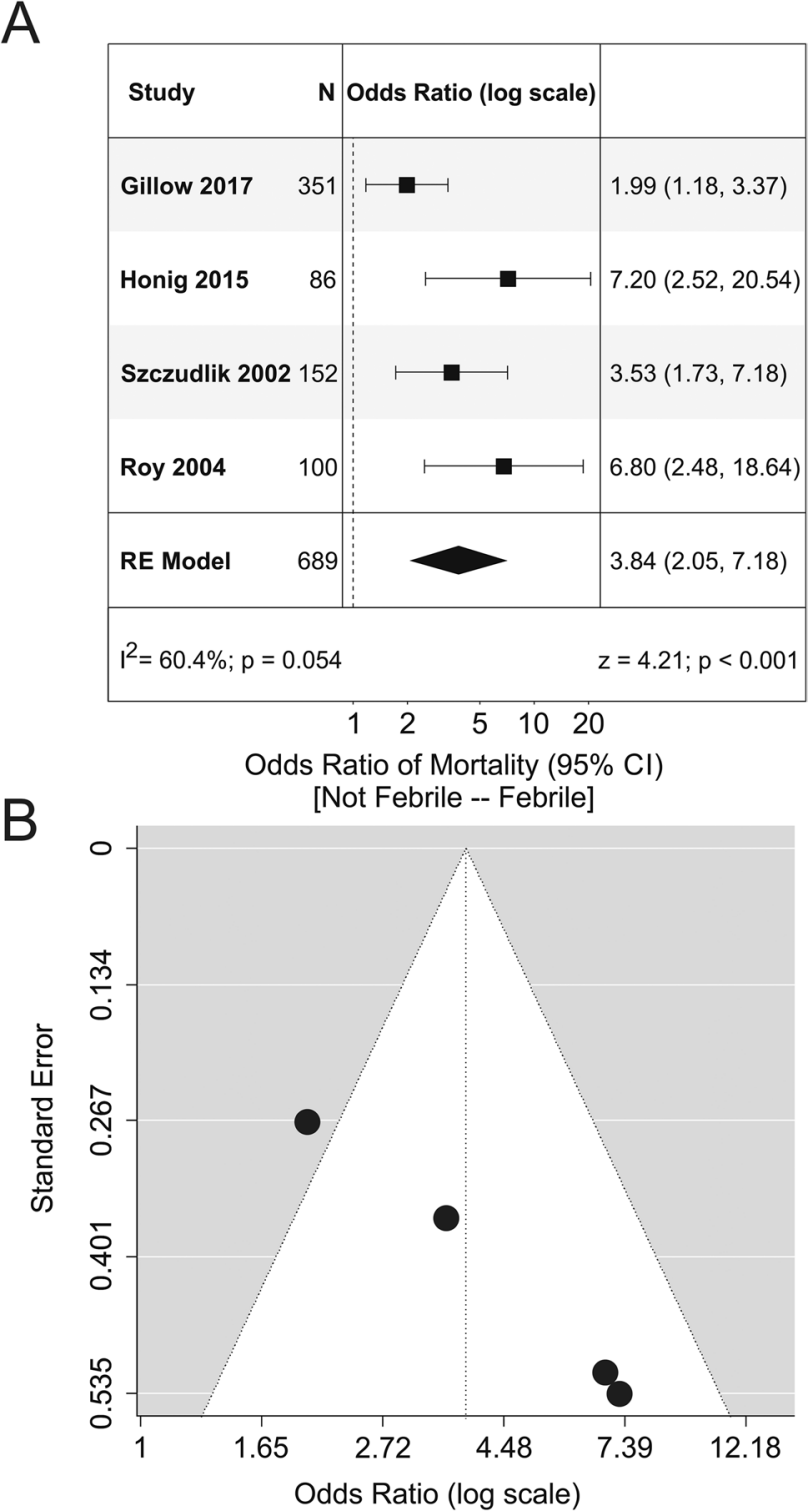
Meta-analysis of mortality related to fever. **A** Forest plot analyzing the odds ratio of mortality in febrile patients. There was no significant heterogeneity between the studies (I^2^ = 60.4%). **B** Funnel plot for the model depicted in Panel **A**. There were no obvious signs of funnel plot asymmetry. RE model, random effects model

**Fig. 3:**
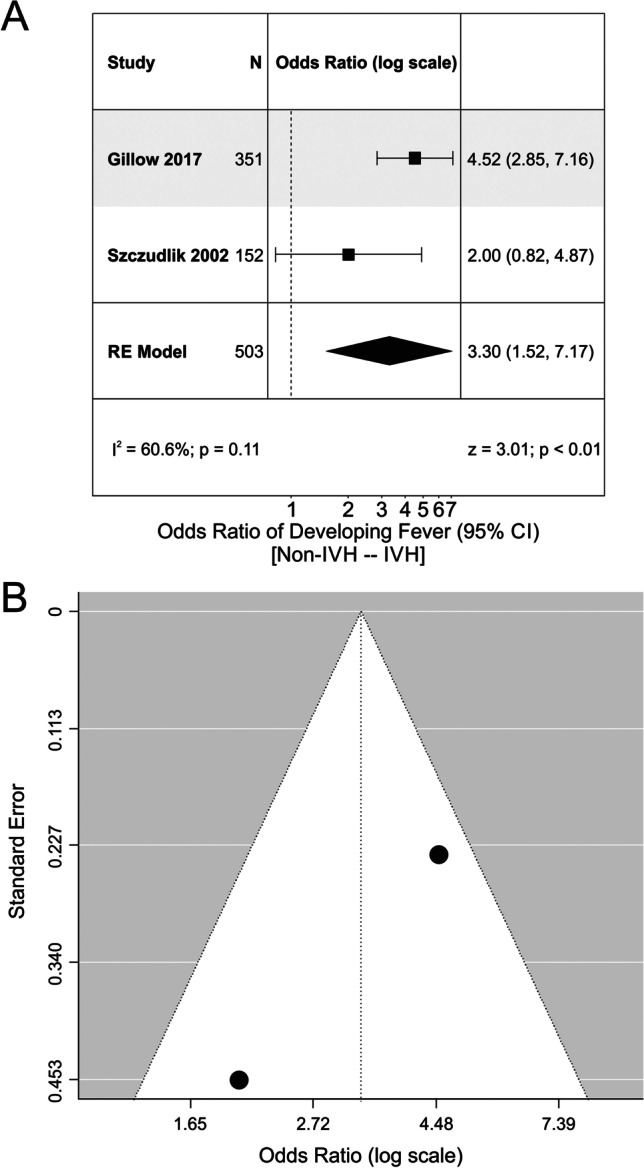
Meta-analysis of fever with respect to ICH location. **A** Forest plot analyzing the odds ratio of developing fever with ventricular extension of the hematoma following intracerebral hemorrhage. There was no significant heterogeneity, though the number of studies was limited (I^2^ = 60.6%). **B** Funnel plot of Panel **A**. No obvious funnel plot asymmetry was detected, but the number of studies that assessed this outcome were limited. IVH, intraventricular hemorrhage; RE model, random effects model

**Fig. 4:**
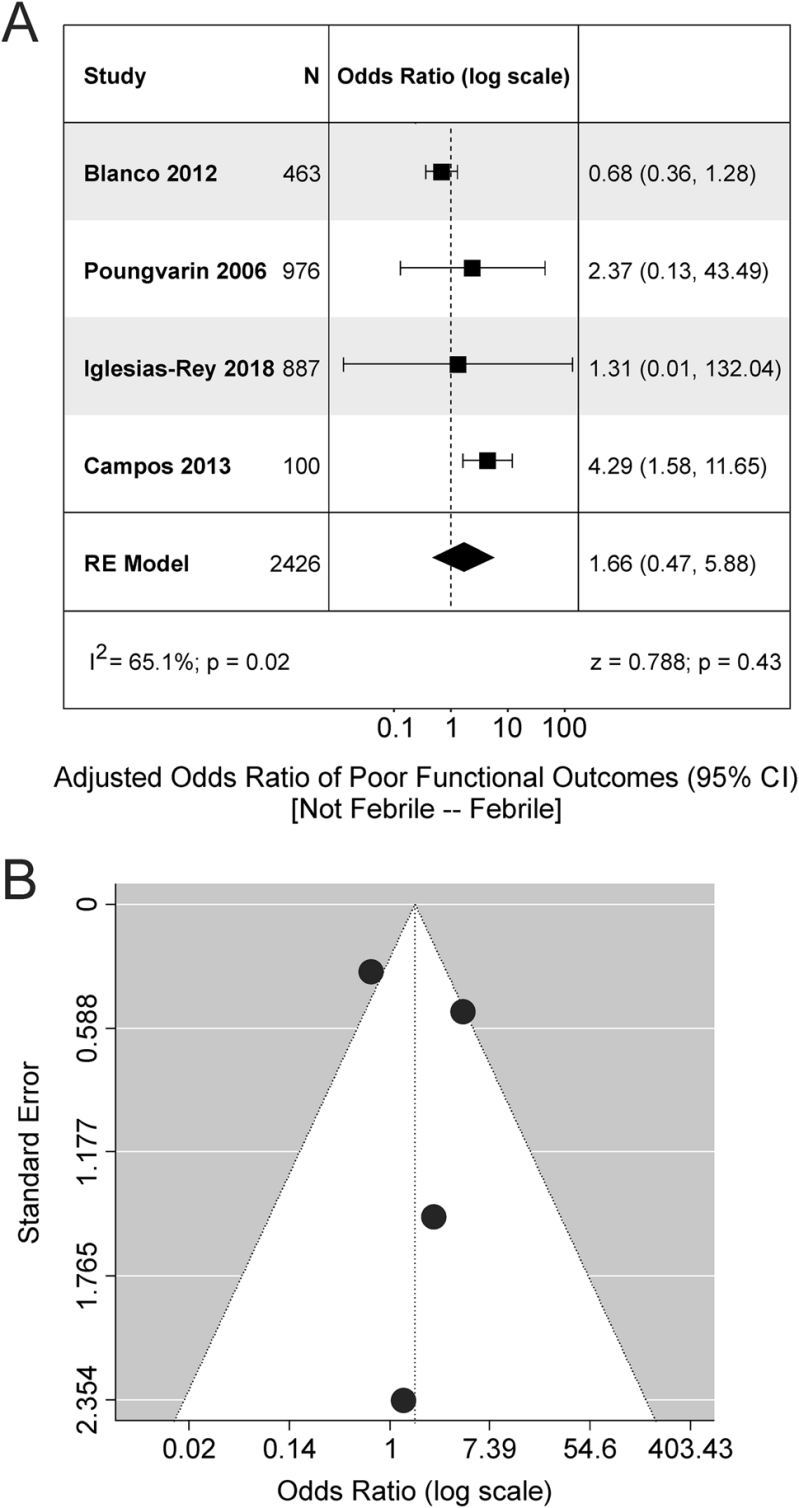
Meta-analysis of functional outcomes following fever in ICH. **A** Forest plot analyzing the odds ratio of poor functional outcomes in febrile patients. The model returned a significant effect. However, there was significant heterogeneity of effect size estimates across studies (I^2^ = 65.1%). **B** Funnel plot depicting the model in Panel **A**. No obvious signs of funnel plot asymmetry were detected. RE model, random effects model

Studies were included in the following analyses if they reported odds ratios, or provided the data required to calculate the odds ratios, of the relationship between an outcome variable and fever/hyperthermia. Multiple studies analyzed mortality, risk of intraventricular extension of the hemorrhage, and poor functional outcomes (defined as mRS > 2), and thus were included in the analysis. Four studies analyzed the relationship between fever and mortality. Overall, there was a significant increase in the odds of mortality if the patient was febrile (OR, 3.84; 95% CI, 2.05 to 7.18; Fig. 2). However, Egger regression revealed signs of funnel plot asymmetry (*p* < 0.01). Additionally, there was a significant increase in the odds of developing fever if the patient had ventricular extension of the hematoma (OR, 3.30; 95% CI, 1.52 to 7.17; Fig. 3). Egger regression could not be completed due to the lack of studies investigating the association between fever and intraventricular hemorrhage. There was no significant relationship between fever and poor functional outcomes in the studies (OR, 1.66; 95% CI, 0.47 to 5.88). Egger regression did not show signs of publication bias (*p* = 0.84) (Fig. 4).

### Literature Related to the Clinical Use of Antipyretics in ICH

Our search returned 10 studies that tested the impact of antipyretic medications in patients (Table 2). Data were not subjected to meta-analysis owing to the great heterogeneity in endpoints, experimental design, and analyses. Only two studies tested the impact of an antipyretic medication against a concurrent conventional care group. Six out of ten studies used multiple medications and/or cooling modalities (e.g., pharmacological and physical cooling). We found that acetaminophen and metamizole were commonly used in tandem to treat fever in patients with various brain injuries (intracranial hemorrhages, ischemic stroke, traumatic brain injury). Eight studies included multiple brain injury subtypes in their inclusion criteria. Of the 8 studies with multiple brain injury subtypes, only 3 provided subgroup analysis of the ICH patient data (i.e., the other 5 studies analyzed the impact of various hypothermia protocols on all brain injury subtypes together). Studies with multiple groups included as few as 2 ICH patients in a treatment arm, up to 87 (median = 19). In general, the findings of these studies suggested that normothermia (especially pharmacologically-induced normothermia) is difficult to maintain as relapse rates are common, and that monotherapies are often not as effective as multifaceted therapies.

**Table 2:** Summary of studies employing antipyretic agents in post-ICH fever analyses (✓ = yes, ✕ = no)

Reference	Antipyretic strategy	Dose (mg)	Rate (hours)	Administration route	ICH patients (N)	ICH subgroup analysis	Temperature measured	Temperature measurement site	Patient population	Primary finding(s) (compared to control groups)
Diringer 2014	Acetaminophen, surface cooling vs. catheter-based normothermia	650	4–6	Oral, rectal	60	✕	✓	N/A	ICH, IS, SAH, TBI	Acetaminophen & surface cooling were ineffective at reducing fever burden compared to catheter-based normothermia
Den Hertog et al. 2009	Acetaminophen vs. placebo control	1000	6	Oral, rectal	173	✓	✓	Tympanic, rectal	ICH, IS	Acetaminophen reduced body temperature by 0.26 °C, with no significant impact on mRS vs. placebo controls
Broessner et al. 2009	Acetaminophen, ibuprofen, surface cooling vs. catheter-based normothermia	500	4	Oral	41	✓	✓	Bladder	SAH, ICH, IS	Acetaminophen group had greater fever burden vs. catheter-based normothermia
Kallmunzer et al. 2011	Acetaminophen, Metamizole, surface cooling vs. historical control group	1000	6	IV	25	✕	✓	Tympanic	ICH, IS	Combined acetaminophen, metamizole, calf packing & saline was more effective than fanning & acetaminophen at reducing fever vs. historical controls
Middleton et al. 2011	Acetaminophen (+ multidisciplinary care) vs. conventional care	N/A	N/A	IV, oral, rectal	51	✕	✓	N/A	ICH, IS	Combined acetaminophen, metamizole, calf packing & saline was more effective than fanning & acetaminophen at reducing fever
Picetti et al. 2014	Acetaminophen & surface cooling	1000	Single dose	IV	2	✕	✓	Bladder, endovascular	ICH, IS, SAH, TBI	Acetaminophen reduced cerebral perfusion pressure and mean arterial pressure
Lord et al. 2014	Acetaminophen, surface cooling vs. historical controls	650	4–6	Oral	80	✓	✓	Bladder	ICH	Acetaminophen reduced temperature (~ 0.5 °C-1°C), increased length of stay, and incidence of hyperglycemia vs. historical controls
De Ridder et al. 2017	Acetaminophen vs. placebo controls	1000	4	Oral, rectal	20	✓	✓	Tympanic, rectal	ICH, IS	Acetaminophen had no effect on temperature, mRS, or adverse events compared to placebo controls
Hervella et al. 2020	Acetaminophen,Metamizole, (retrospectively dichotomized into response categories)	1000	8	Oral	795	✓	✓	Axillary	ICH	Acetaminophen increased mRS scores and decreased perihematomal hypodensity in responders vs. non-responders
Lee et al. 2021	Acetaminophen, Metamizole, surface cooling vs. conventional care	1000	6	IV	16	✕	✓	Tympanic	ICH, IS, SAH	Combined acetaminophen, metamizole, calf packing reduced fever duration vs. historical conventional care group

*IV* intravenous, *SAH* subarachnoid hemorrhage, *TBI* traumatic brain injury, *IS* ischemic stroke, *ICH* intracerebral hemorrhage, *mRS* Modified Rankin Scale

## Discussion

Fever, or excess heat production, is one of the 5 cardinal symptoms of inflammation [16, 39]. Fever is thought to develop through changes in the activity of the brain’s thermoregulatory centers (e.g., hypothalamus) [13]. The most well-studied pathway for fever development describes fever after peripheral injury and/or infection (for a detailed review, see [40]). As pyrogens (e.g., cytokines, bacterial, fungal, or viral molecules) reach the organum vasculosum of the lamina terminalis, they stimulate the production of intracerebral prostaglandin E2 (PGE2), which can lead to a febrile state [41], including when administered after ICH where it appears to worsen outcome in rat, but not mouse where PGE2 appears to be beneficial [42]. PGE2 acts on the preoptic area of the anterior hypothalamus [43] and is known to decrease the activity of warm-sensitive cells and increase the activity of cold-sensitive cells there, resulting in increased cellular metabolism, thermogenesis, immune cell activation, and vasoconstriction [44, 45]. PGE2 is also partially responsible for some of the cardinal symptoms that accompany fever (e.g., redness, swelling, and pain) and thus may be a therapeutic target following ICH [46].

There are two general phases of inflammation that must occur for optimal post-ICH recovery. First, there is removal of the hematoma and injured tissue via microglial activity and recruitment of peripheral macrophages, and following this, the resolution and repair phase. In rodents, the removal phase begins quickly, with neutrophil infiltration occurring within hours [47] and peaking by day 3 post-ICH [48]. Microglial activity appears to be more drawn out, with studies demonstrating rapid activation, with peak activity between days 3 and 7, and continued activity for up to 4 weeks [49, 50]. Clinical studies have shown a similar profile of neutrophil and microglial activity [51, 52]. There is evidence that blocking elements of the inflammatory response may limit post-ICH recovery by delaying hematoma removal, thereby resulting in progressive neurotoxic injury [53, 54]. Moving forward then, we must clarify when and which elements of fever are injurious.

Compared to ICH, research in cerebral ischemia has more clearly established the prognostic significance of fever [6], highlighted many of the mechanisms through which fever worsens outcome [55, 56], and nicely complimented the preclinical ischemia literature, where post-stroke temperature increases alone were sufficient to worsen injury [57–59]. In ischemic stroke, fever worsens secondary injury through a heightened inflammatory response and hypermetabolism that follow an increased body temperature [60]. Additionally, increased brain temperatures and inflammatory activity result in increased blood brain barrier permeability, greater recruitment and activation of leukocytes, accelerated gliosis, greater free radical production, and acidosis [61–65]. Other molecular mechanisms that are associated with poorer outcomes in febrile ischemic stroke patients include overexpression of intracellular adhesion molecule 1, interleukin 6, tumour necrosis factor alpha, C-reactive protein, glutamate, and more [61, 66]. Each of the aforementioned processes and mechanisms appears to be accelerated by fever, and is associated with greater secondary injury after ischemic stroke. In the context of ICH, overactivity in these processes and elevated levels of these markers are associated with poorer outcomes, but little data exist with a focus on the impact of fever and outcomes related to these markers [67–69].

Our meta-analysis suggests that patients may be more likely to develop fever if they also have ventricular extension of the hematoma. However, only two studies provided data that could then be used in the meta-analysis, and thus, future investigators may wish to include data related to the relationship between fever and intraventricular hemorrhage in their publications. Regardless, these data are not surprising, as investigations related to the role of intracranial bleeding and fever development have been published almost a century ago [70, 71]. Our meta-analysis also demonstrated an increased probability of mortality for febrile ICH patients, compared to non-febrile ICH patients, but no effect on poor functional outcomes. Importantly, the functional outcome data must be interpreted by bearing in mind that mortality was increased in febrile patients, which may act as a confound by causing selective attrition, thereby making it difficult to discern a negative effect on functional outcome. Next, we found that most clinical studies provided supporting evidence for a harmful role of post-ICH fever; however, these studies often left out critical information (e.g., location of temperature monitoring, fever incidence values, fever treatment protocols, etc.), which may contribute to the heterogeneous findings that limit future interpretation and synthesis (e.g., meta-analysis). Regarding fever management, there is a need to develop and implement discrete, stroke-specific, protocols surrounding the treatment of fever specifically in ICH patients, as many protocols encompass multiple neurological injuries. In other words, these broad fever treatment protocols imply that fever is equivalently harmful across a diversity of brain injuries, and additionally may assume that of febrile responses as well (these assumptions are yet to be tested, as sufficient data are not available). Disease-specific protocols are especially important because we found that an average of 35% of ICH patients are febrile, and given the risk of ventricular extension of the hematoma, neurogenic fever appears to have a higher prevalence and likely a differential treatment approach.

Current guidelines suggest that fever may be harmful after ICH and that maintaining normothermia may be reasonable, though the evidence base is limited (Class IIb, Level of Evidence C; [6]). These recommendations are based on inconsistent associational evidence in ICH, as we have reviewed here, and are often borrowed from literature on other brain injuries, such as ischemic stroke, where the evidence is more convincing [72]. Although pathophysiological mechanisms overlap, ICH and cerebral ischemia have distinct post-stroke mechanistic profiles [73, 74], which explains why it is not uncommon for treatments that work in ischemia to fail in ICH (e.g., hypothermia in animal models; [75, 76]). Moreover, ICH subtype is rarely considered in fever research, and temperature may have more prognostic significance in some ICH subtypes or locations compared to others, such as in hypertensive ICH compared to cerebral amyloid angiopathy-related ICH [77]. Our analysis of clinical literature has shown that post-ICH fever is associated with higher mortality but not worse functional outcomes. However, the relationship between temperature and outcome is complex and may depend on a multitude of factors, such as the timing and duration of temperature increase, magnitude of temperature change, fever origin, and rate of return to baseline temperature. The most common predictors of poor outcomes in ICH are hematoma size, growth, and location [21], each of which are established predictors of ICH-related mortality and morbidity. Thus, studies must carefully exclude such third variables to strengthen and clarify the relationship between fever and post-ICH outcomes. Particularly, evidence suggests that the relationship between fever and hematoma growth may be bidirectional [25]. Just as higher temperatures may lead to an increase in stroke size, larger strokes may lead to higher temperatures. This may be partially due to larger strokes damaging thermoregulatory brain structures, which may occur with intraventricular extension of a hematoma causing hypothalamic injury. It is important to note that hyperthermia did not worsen bleeding in the rat using either the collagenase or autologous whole blood models of ICH [30, 31]. Additionally, there is no clear linear relationship between organismal temperature and post-ICH outcomes. Therefore, we must question whether the relationship between fever and post-ICH outcomes is linear and unidirectional, or of some other nature. Perhaps, this relationship is complicated, such that temperature increases up to a certain threshold are inconsequential, or perhaps beneficial, with greater temperature increases becoming dangerous [77, 78]. This concurs with our current conceptualization of the role of temperature increases after injury and infection, as the activity of immune cells and factors are potentiated, and bacterial proliferation is impaired at elevated temperatures [13].

As noted, animal hypo- and hyperthermia studies suggest that ICH-related outcomes are relatively temperature insensitive [79]. Indeed, work from our laboratory found that inducing hyperthermia did not worsen brain injury, bleeding, or functional outcomes in either of the common ICH models in rats [30, 31]. Although they did not include a cerebral ischemia plus hyperthermia group as a positive control, the hyperthermic temperature treatments that were administered are known to be devastating after cerebral ischemia [58]. These are the only animal studies of post-ICH temperature increases that we are aware of. Importantly, fever rarely occurs in widely used animal ICH models, although it should be noted that many do not monitor post-ICH temperature, so fever from any cause (e.g., surgical infection) could easily be missed, and no animal studies have examined whether treating fever is of benefit. However, fever can be induced, but more work is needed to determine the impact of this. For example, we recently found that a modified collagenase-induced medial striatal ICH with ventricular extension caused an early, monophasic neurogenic fever (histologically confirmed hemorrhagic extension into the ventricular system) with a 24-h duration (Liddle, Dirks, and Colbourne, unpublished data). In preclinical studies, the extent to which induced hyperthermia is similar to true fever is currently unknown in preclinical ICH research, serving as a key limitation and knowledge gap, these data would undoubtedly prove to be useful for future investigations.

Clinically, few studies examined mechanisms by which hyperthermia or fever affect outcomes, and Table 2 demonstrates that few antipyretic studies exist in ICH. In particular, only two studies, the Paracetamol [Acetaminophen] In Stroke (PAIS II and III) trials, used concurrent control groups and provided ICH subgroup analyses to test whether antipyresis is beneficial in the context of ICH. Unfortunately, the PAIS II and III trials were underpowered, but demonstrated a small impact of antipyresis via acetaminophen on body temperature reduction, and no benefit on mRS compared to placebo controls. Based on the current state of evidence, it is unclear if, and when, fever treatment would be beneficial after ICH, especially with high rates of fever relapse, varying fever treatment protocols, and few rigorous, high-powered studies on this topic.

The use of pharmacological agents to prevent and/or treat fever after ICH (e.g., anti-inflammatory drugs or antibiotics) has associated risks that should be considered. While recent literature suggests that acetaminophen is safe to use as an antipyretic therapy in other brain injuries [13], its use has not been extensively explored after ICH, either in the clinic or animal models. Indeed, Table 2 suggests that very few studies have been employed on the topic of antipyresis in ICH, which makes data synthesis and interpretation difficult. As a result of this, current guidelines rely heavily on evidence from other brain injuries, which often employ low doses of acetaminophen, which produce few to no benefits in some cases [13]. Thus, larger doses may be required; however, this route would put ICH patients, many of which already present with other comorbidities (e.g., hypertension, diabetes) at further risk for complications (e.g., renal and hepatic injury, further cerebrovascular events) [80, 81]. Studies on other anti-pyretic therapies have also been investigated (e.g., cyclo-oxygenase inhibitors), but no conclusions on their use, pertaining to fever specifically, have been drawn [82]. Antibiotics have also been shown as effective agents in preventing and treating post-stroke fever [83, 84]. However, the impact of post-stroke prophylactic antibiotics on functional outcomes and mortality remains unclear and should be approached cautiously [85]. In sum, our understanding of post-ICH fever therapies is lacking, but the data suggest that differentiating between fever types (e.g., neurogenic or infectious) and discerning the impact of the temperature response itself is critical, and well-timed, targeted therapies may be advantageous to broad spectrum treatments.

## Limitations

Many of the limitations in our analyses are the result of a paucity of studies on the topic of fever in ICH. For example, we did not have a sufficient number of studies or granularity in our data to directly assess the relationship between study variables and outcomes with meta-regression techniques. Additionally, some planned a priori analyses could not be completed, owing to limited data (e.g., Egger regression). Further, we chose random effects meta-analysis as a more conservative method to combine odds ratios and determine model significance across studies, further decreasing statistical power to detect potential fever-related effects in the context of few studies. And lastly, we could not analyze changes in fever-related mechanisms using meta-analytic techniques owing to limited investigations on this topic. Ultimately, the data herein will serve as a foundation and call to action for future analyses.

## Recommendations and Future Directions

There is a dearth of clinical literature examining temperature increases in the context of ICH. Published data are limited in that important study parameters are often difficult to obtain. Furthermore, data are often pooled across brain injuries, making ICH-specific interpretations difficult or impossible. These limitations present future investigators with an opportunity to design experiments that are consistent with clinical recommendations for ICH researchers (e.g., the HEADS recommendations) [74]. In line with the HEADS participants, we recommend that studies include systematic, standardized methods, endpoints (e.g., long-term outcome), and analyses that are developed a priori and have sufficient statistical power.

Open science may solve some of the problems that we have highlighted, given that experimental design and data collection standards are followed. Often, data reporting is suboptimal as it is not standardized, and publishers often have strict word counts [86]. Data sharing and open access would allow investigators to take a more comprehensive approach to data exploration. With open science and data sharing, researchers can easily make cross-study comparisons. In addition, statistical models could be explored for known mediators of the relationship between ICH and outcomes, and re-analyzed using standardized model parameters. Finally, increased data accessibility would allow for meta-analysis of data to be more easily conducted [87]. At this time, there are no meta-analyses on the role of post-ICH fever in animals or patients.

Preclinical studies are necessary to further our mechanistic understanding of post-ICH fever. Animal models allow for high internal control, and manipulation of variables that cannot be easily addressed in clinical settings. Unfortunately, post-ICH fever models are scant and use hyperthermia to represent fever, with only two preclinical studies existing to our knowledge. Accordingly, researchers may wish to investigate novel, preclinical models of fever that more accurately represent the etiology and pathophysiology of both neurogenic and infectious fever.

## Conclusion

We found that fever increases mortality following ICH but may not affect functional outcomes, with intraventricular hemorrhage appearing to increase the incidence of fever. Studies directly investigating the role of fever in ICH are limited, with much of the literature consisting of mixed, associational evidence and limited understanding of mechanisms of injury or therapeutic targets. Such limited data has led to the adoption of findings from related brain injuries (e.g., cerebral ischemia), which may not translate to ICH. Current guidelines suggest that post-ICH fever is deleterious and should be prevented by maintaining normothermia. These guidelines are based upon a limited, and likely confounded evidence base in ICH, and this clinical literature is not yet conclusive owing to numerous issues. Thus, we conclude that broadly labelling fever as detrimental is a critical limitation in the current literature and suggest that a more comprehensive and nuanced approach be taken to the study and treatment of fever in ICH. Furthermore, there are only two preclinical studies modelling post-ICH temperature increases, and these do not support clinical assumptions that raised temperature is harmful, at least within the range they tested in rats and commonly seen in patients. Intuitively, it may be that the etiology of the fever is more important than the fever per se (i.e., appropriate treatment of infectious fever, and removal of the hematoma from the ventricular system and hypothalamus). In the greater literature, less is known about neurogenic fever, and thus, comprehensive studies are needed to better understand whether pathophysiological concepts in infectious fever apply to fever that is neurogenic in nature. Ultimately, the role that fever-related mechanisms play in post-ICH injury is largely unknown, and future studies should be undertaken to better understand the role of fever in ICH.
